# Seasonal variations in composition and function of gut microbiota in grazing yaks: Implications for adaptation to dietary shift on the Qinghai‐Tibet plateau

**DOI:** 10.1002/ece3.70337

**Published:** 2024-10-22

**Authors:** Xungang Wang, Tongqing Guo, Qian Zhang, Na Zhao, Linyong Hu, Hongjin Liu, Shixiao Xu

**Affiliations:** ^1^ Key Laboratory of Adaptation and Evolution of Plateau Biota Northwest Institute of Plateau Biology, Chinese Academy of Sciences Xining China

**Keywords:** adaption, gut microbiota, microbial function, seasonal shift, yak

## Abstract

Gut microbiome of animals is affected by external environmental factors and can assist them in adapting to changing environments effectively. Consequently, elucidating the gut microbes of animals under different environmental conditions can provide a comprehensive understanding of the mechanisms of their adaptations to environmental change, with a particular focus on animals in extreme environments. In this study, we compared the structural and functional differences of the gut microbiome of grazing yaks between the summer and winter seasons through metagenomic sequencing and bioinformatics analysis. The results indicated that the composition and function of microbes changed significantly. The study demonstrated an increase in the relative abundance of Actinobacteria and a higher ratio of Firmicutes to Bacteroidetes (F/B) in winter, this process facilitated the adaptation of yaks to the consumption of low‐nutrient forages in the winter. Furthermore, the network structure exhibited greater complexity in the winter. Forage nutrition exhibited a significant seasonal variation, with a notable impact on the gut microbiota. The metagenomic analysis revealed an increase in the abundance of enzymes related to amino acid metabolism, axillary activity, and mucin degradation in the winter. In conclusion, this study demonstrated that the gut microbiome of grazing yaks exhibits several adaptive characteristics that facilitate better nutrient accessibility and acid the host in acclimating to the harsh winter conditions. Furthermore, our study offers novel insights into the mechanisms of highland animal adaptation to external environments from the perspective of the gut microbiome.

## Graphical abstract

The composition and function of gut microbiota in grazing yak differed significantly between the summer and winter. Seasonal dietary shift impacted microbial communities' composition and function. Grazing yak adapts to the harsh winter environment via specific gut microbiota.

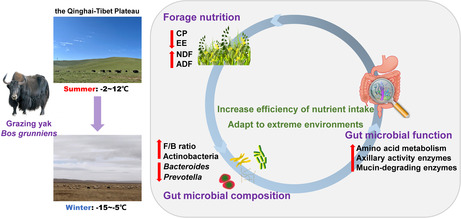

## 1 INTRODUCTION

A large number of microorganisms inhabits the intestines of humans and animals, coevolving to establish a distinctive microbial ecosystem during host growth and developmental processes (Kinross et al., 2011). These microbe communities coexist and interact with animal hosts, creating a tightly integrated unit that is known as the “second genome” of the host (Grice & Segre, 2012). The gut microbiota plays an important role in various physiological activities, including food digestion, energy metabolism, nutritional homeostasis, immune function, and host development (Ley et al., 2006; Liu et al., 2022; Sonnenburg & Bäckhed, 2016; Thaiss et al., 2016). The diversity, composition, and function of the gut microbiota are influenced by a range of factor such as host diet, age, sex, season, habitat, and other external environmental variables (Aricha et al., 2021; Li et al., 2021; Ren et al., 2017; Wang, Hu, et al., 2021; Wang, Jin, et al., 2021). Should these factors undergo any alterations, the dynamic equilibrium of the gut microbiota will become imbalanced.

Previous studies have indicated that the diet of host plays a significant role in determining the diversity and composition of the gut microbiota in animals (Muegge et al., 2011; Rothschild et al., 2018). For example, seasonal dietary shifts drive a notable shift in the composition and function of gut microbiomes in the great evening bat, and these alterations were linked to heightened energy requirements for hunting and storing fat for hibernation and migration (Gong et al., 2021). Additionally, the gut microbiota composition of *Pomacea canaliculata* varied significantly across the summer, autumn, and winter seasons (Li et al., 2022). Phenological alternations in seasonal food availability can also affect the gut microbial diversity and community structure of white‐lipped deer (You et al., 2022). Understanding the gut microbial diversity, composition, and function of animals can provide a comprehensive understanding of the mechanisms of their adaptation to environmental change, particularly in the examination of high‐altitude animals.

The yak (*Bos grunniens*) is a distinctive ruminant herbivore inhabiting high‐altitude regions, primarily within the Qinghai‐Tibet Plateau, where it thrives at altitudes exceeding 3000 m (Ding et al., 2014). It can adapt well to high‐altitude environments characterized by low temperatures, reduced oxygen levels, strong ultraviolet radiation, and limited food resources (Qiu et al., 2015). In the conventional grazing system, yaks depend mainly on the natural herbage present in alpine meadows for sustenance and do not receive any supplementary feed. However, the herbage available during the extended cold season is inadequate, resulting in malnutrition, health related problems, and a decrease in live‐weight (Long et al., 2008; Zhao et al., 2018). In spite of these challenging environmental conditions, yaks have adapted well and flourished for thousands of years, sustaining their population. Mounting studies have shown that the gut microbiota of grazing yaks allows them to adapt more efficiently to food resources with different nutritional compositions, ensuring that their own nutritional requirements and utilization are met (Guo et al., 2021; Ma et al., 2019). When yaks shift from grazing to being house‐fed, the gut microbiota community structure undergone significant changes (Zhang et al., 2020). However, there is currently a relative lack of research on the gut microbial function of grazing yaks, especially using metagenomic‐sequencing technology.

Here, this study investigates the seasonal variations in gut microbiota composition and function of grazing yaks on the Qinghai‐Tibet Plateau with metagenomic sequencing. We hypothesize that significant variation in gut microbiota composition and function in yaks occurs between summer and winter, and these changes could help yaks better adapt to the harsh environment in winter. This study provides new insights on how the gut microbiota adapts to seasonal and dietary variations, and offers a scientific basis for the health assessment and extreme environmental adaptation of highland animals.

## 2 MATERIALS AND METHODS

### 2.1 Fecal sample collection

**FIGURE 1:**
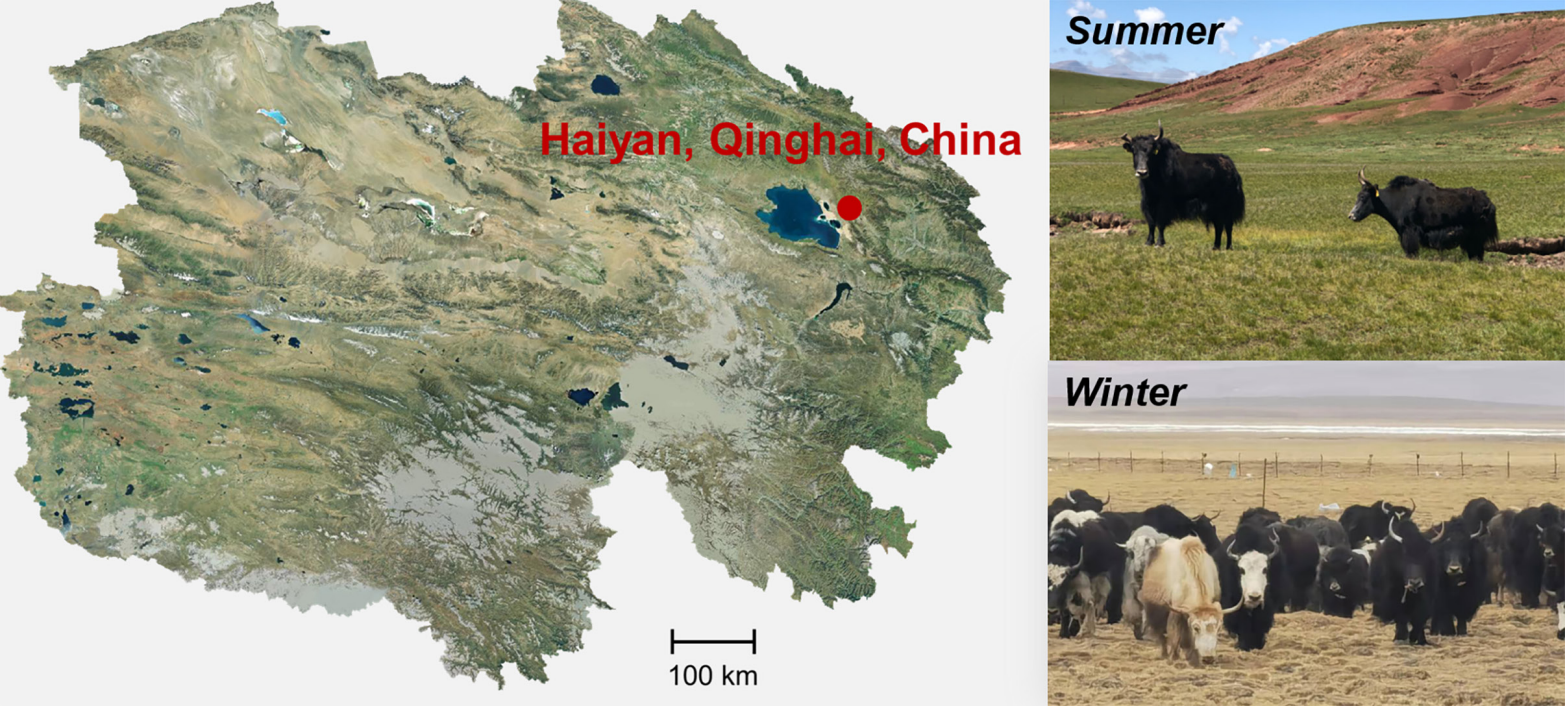
The sampling site and photos of grazing yaks.

Grazing yak fecal samples were collected during both summer (July) and winter (December) seasons, in Haiyan County (altitude: 3150 m; 36°55′ N, 100°57′ E), located in Qinghai Province, China (Figure 1). Eight two‐year‐old healthy male yaks with similar body weights (123.60 ± 11.96 kg) were labeled and grazed with livestock crowd on the fixed area pasture without feed supplementation from July, grazing activities usually lasted from 08:30 to 17:30, then entered shelter for overnight (Xu et al., 2017). The plant community of natural pasture is dominated by *Kobresia humilis*, *Leymus secalinus*, *Elymus nutans*, *Stipa purpurea*, and *Carex aridula* (Wang, Wu, et al., 2023; Wang, Zhang, et al., 2023; Yang et al., 2019). In December, the labeled eight yaks were weighted again (body weight: 117.40 ± 8.04 kg). Feces were collected from each selected yak by direct rectum grab sampling according the method of Franco‐Lopez et al. (Franco‐Lopez et al., 2020). Briefly, the rectal wall was massaged to stimulate rectal evacuation, and the resulting feces were collected. The samples were immediately frozen in liquid nitrogen and stored at −80°C for subsequent analysis.

### 2.2 Determination of forage nutrition composition

The grazing pasture was sampled using sample squares (0.5 m × 0.5 m) that were randomly positioned in the center of the field, with six samples collected at a time in which the pasture was cut to approximately 2 cm above the ground. All collected diets were oven dried at 60°C for 48 h and individually ground with a grinder to pass a 1 mm sieve for nutritional quality determination. The dry matter of the pasture in summer and winter was 94.65% and 97.34%, respectively. The crude protein (CP) and ether extract (EE) were quantified according to the AOAC methods (AOAC, 2000); the neutral detergent fiber (NDF) and acid detergent fiber (ADF) contents were determined according to Van Soest et al. (Van Soest et al., 1991).

### 2.3 Metagenome sequencing and annotation

A total of sixteen samples (summer: *n* = 8; winter: *n* = 8) were selected for metagenomic sequencing. Genomic DNA was extracted from the fecal samples using the genomic DNA extraction kit from TIANamp (TIANGEN). The DNA concentration and integrity were assessed with Nanodrop 1000 (Thermo Fisher Scientific) and 1% agarose gel. Metagenomic DNA libraries were constructed using the TruSeq DNA Sample Prep kit (Illumina) and were sequenced by Illumina PE150 at Biomarker Technologies. After sequencing, raw reads were filtered using Trimmomatic v0.33 with length <30 bp or with a quality value (<20). The reads considered from the host were removed using bowtie2 v2.2.4, with the *Bos grunniens* genome sequences as references. Contigs of 300 bp and over were selected as the final assembly result (Li et al., 2015). Results were evaluated by Quast v2.3 (Gurevich et al., 2013) and then contigs were used for further gene prediction and annotation. Then, MetaGeneMark v3.26 (Zhu et al., 2010) was used to predict the presence of open reading frames (ORFs). Predicted genes from all samples were gathered together to form a large gene set. MMseqs2 v11‐e1a1c was used to construct the NR gene set by setting 95% identity and 90% coverage of the gene with the longer sequences. Transcripts per kilobase of exon model per million mapped reads (TPM) of the gene, calculated as [(gene count/gene length) × 10^6^/sum (gene count/gene length)], were used to normalize gene abundance. The abundance of genes was compared using the R program DESeq2 package (Love et al., 2014). The Diamond v0.9.29.130 was used to blast unigenes to Non‐Redundant Protein Sequence (NR) Database of the NCBI. The Kyoto Encyclopedia of Genes and Genomes (KEGG) and the Carbohydrate‐Active Enzymes (CAZy) database were also annotated.

### 2.4 Statistical analysis

Statistical analyses were performed using R v4.3.1. The alpha diversity, beta diversity, differences in the relative abundance of major microbial taxa and functional pathways were calculated using the R package (“vegan” and “ade4” packages). Pearson correlation coefficient of the top 30 bacterial genera zoo were calculated using R software (package “igraph”) (Person's correlation greater than 0.8 or lower than −0.8), and then the co‐occurrence network was visualized by Gephi V0.9.2. The paired t‐test was used to compare microbial communities between two groups. Statistical significance was declared at *p* < .05 “*”, *p* < .01 “**”, and *p* < .001 “***”.

## 3 RESULTS

### 3.1 Metagenome sequence statistics

Of all the 16 yak fecal samples, a total of 170,701 megabases (Mbp) clean data persisted after quality control. In total, 153,799 Mbp optimized data and 1,031,994,244 reads were obtained after the elimination of host sequences (*Bos mutus*) (average of 121,411,087 reads per sample), accounting for 90% of the clean reads. A total of 16,678,583 contigs (average of 1,042,411 contigs per sample) from the gene assembly were collected and 12,072,072 genes were identified after gene prediction (Table S1). All these data revealed no statistically significant seasonal variations (*p* > .05). The number of genes shared by warm and cold season is 1,077,063. A total of 6,201,135 genes and 4,793,874 genes were unique to the summer and winter group.

### 3.2 Differences in gut microbial community diversity and composition

**FIGURE 2:**
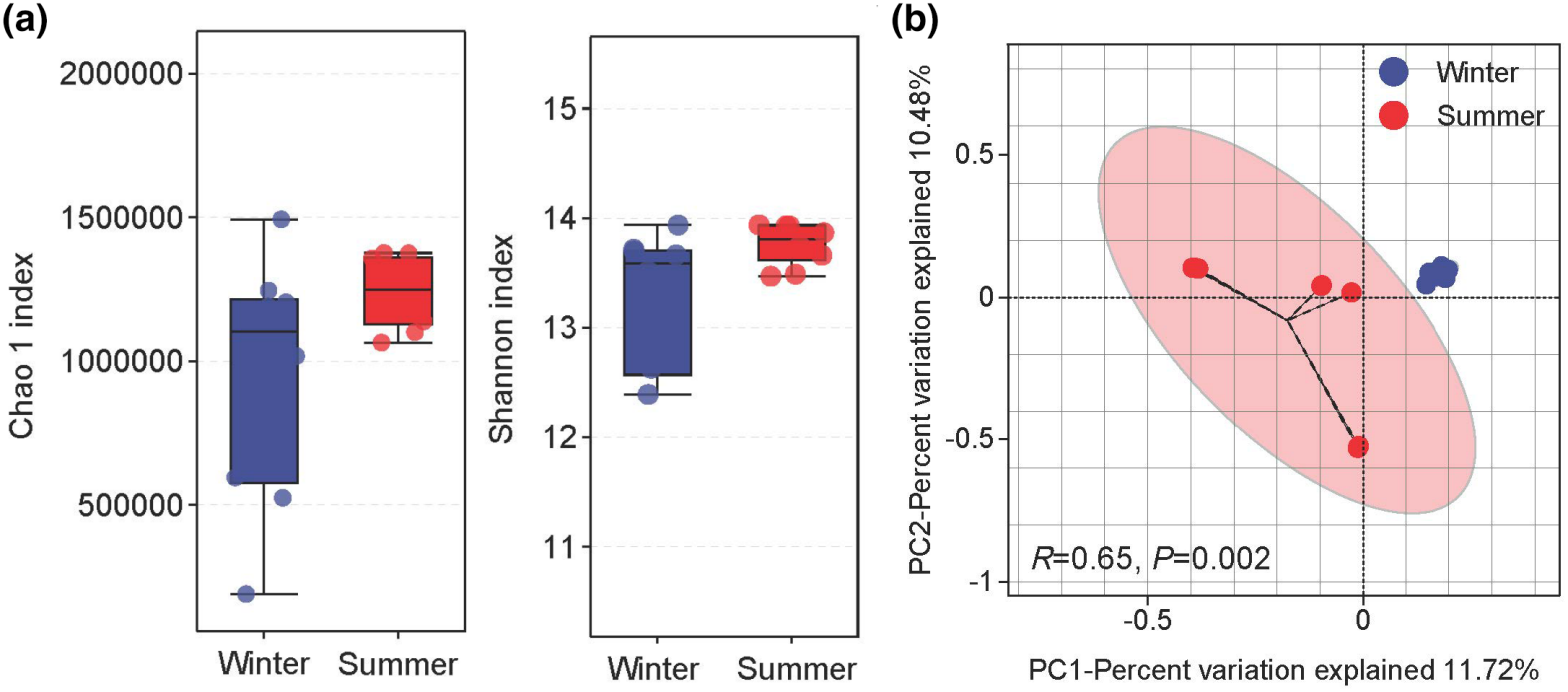
(a) The alpha diversity of the gut microbiome in yaks between different seasons. (b) PCoA based on Bray‐Curtis distances of the gut microbiome in yaks between different seasons.

There were no significant (*p* > .05) differences in the Chao 1 and Shannon indices observed between the summer and winter seasons (Figure 2a). A principal co‐ordinate analysis (PCoA) based on the Bray‐Curtis distances revealed a significant separation between the distribution of fecal samples from the two seasons, which was confirmed by Adonis analysis (*R* = 0.65, *p* = .002) (Figure 2b).

**FIGURE 3:**
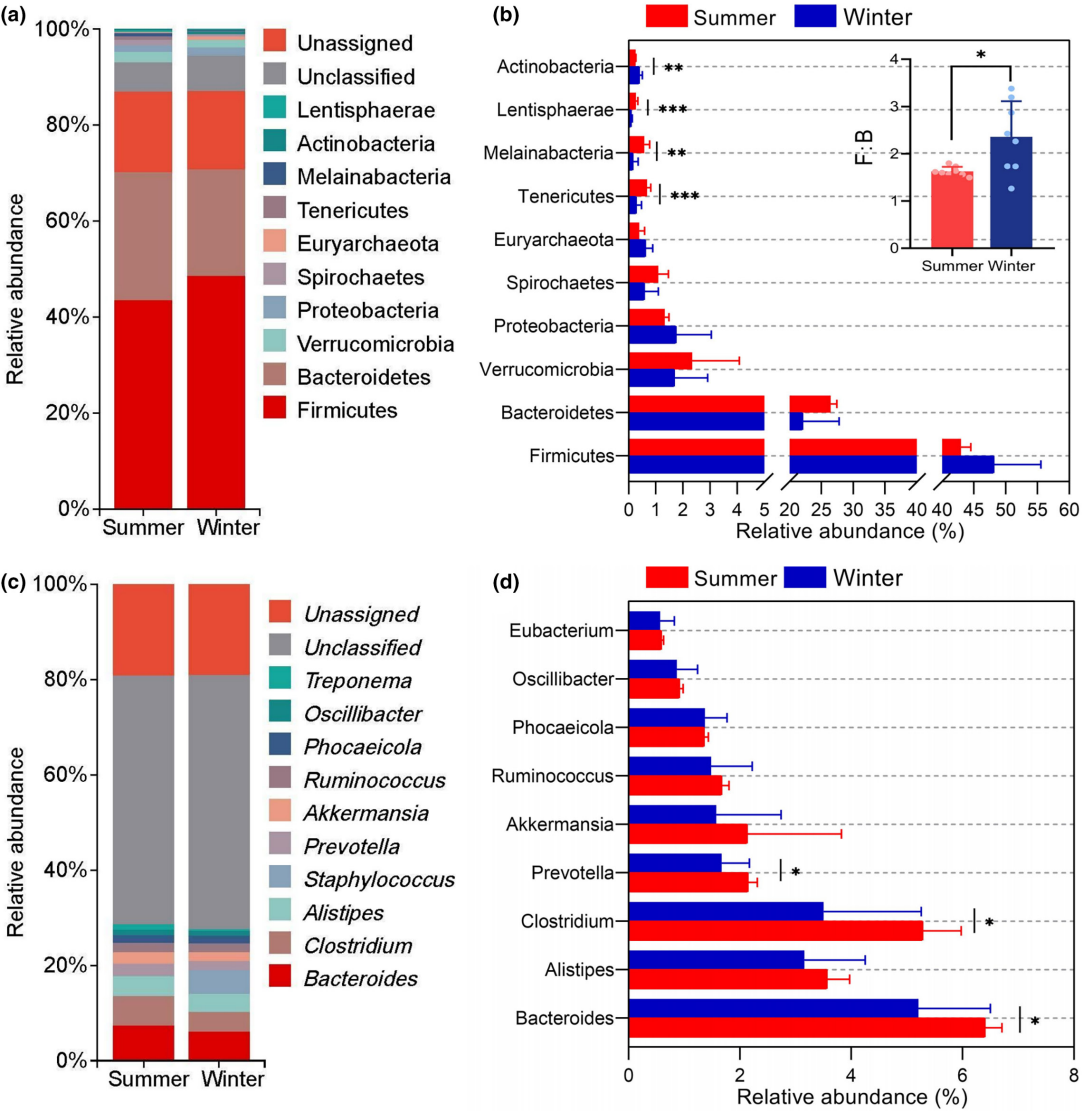
Differences in relative abundance of major phyla (top 10) and major genus (top 10) between seasons. (a) Taxonomic compositions at phylum level. (b) Seasonal differences in relative abundance of major phyla. (c) Taxonomic compositions at genus level. (d) Seasonal differences in relative abundance of major genus. Differences are denoted as follows: *p < .05; **p < .01; ***p < .001.

The metagenomic sequencing results showed that at the kingdom level, the relative abundance of bacteria, archaea, viruses, and fungi was 82.34%, 0.61%, 0.23%, and 0.05%, respectively, with the remaining sequences comprising 16.77% (Table S2). For bacteria, at the phylum level, Firmicutes, Bacteroidetes, Verrucomicrobia, and Proteobacteria were the dominant phyla, with a relative abundance of over 1% (Figure 3a). At the genus level, *Bacteroides*, *Alistipes*, *Clostridium*, *Prevotella*, *Akkermansia*, *Ruminococcus*, and *Phocaeicola* were the most prevalent genera (with a relative abundance >1%) (Figure 3c).

Concurrently, we investigated the differences between the groups of these phylum and genus. At the phylum level, we found that the Tenericutes, Melainabacteria, and Lentisphaerae had significantly higher relative abundance (*p* < .05) in the summer, whereas the winter group demonstrated a higher proportion of Actinobacteria (Figure 3b). Additionally, the results showed that the ratio of the relative abundance of Firmicutes to that of Bacteroidetes (F/B) values was 1.627 ± 0.098 in the summer and 2.358 ± 0.754 in the winter group. Analysis of the differences between the two groups revealed that the F/B value in the winter group was significantly (*p* < .05) higher than that in the summer group. At the genus level, the summer group exhibited a significantly higher relative abundance of *Bacteroides*, *Clostridium*, and *Prevotella* compared to the winter group (*p* < .05) (Figure 3d).

### 3.3 Seasonal differences in gut microbiota co‐occurrence networks

**FIGURE 4:**
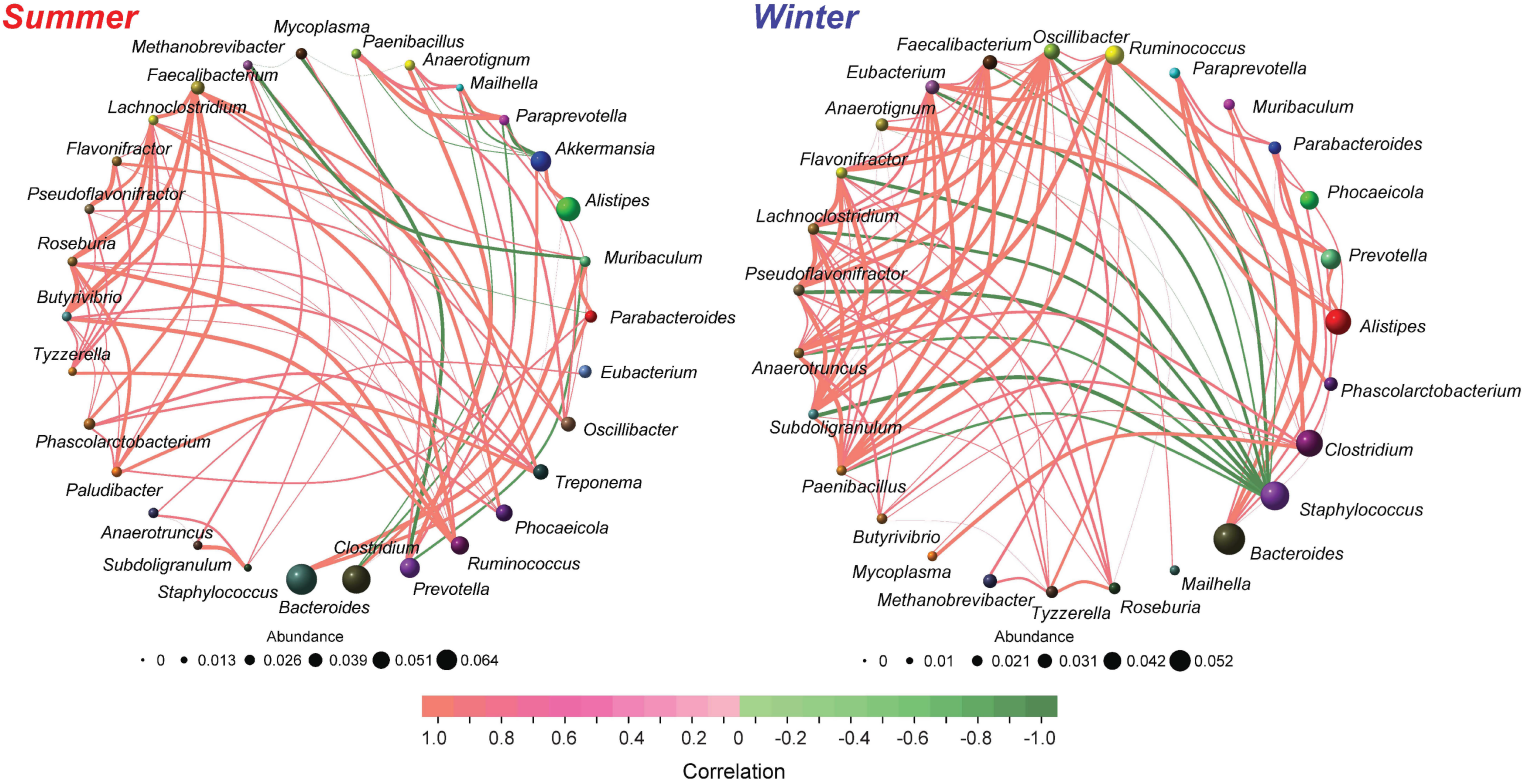
Co‐occurrence networks of the top 30 genera among seasons. Nodes represent genus and their sizes indicate different relative abundance. Links between the nodes represent a significant and strong correlation between two genera (Person's correlation greater than 0.8 or lower than −0.8). Line color reflects direction (green: Negative; orange: Positive).

Based on the results of metagenomic sequencing, the co‐occurrence network analysis was conducted on the top 30 genera of different groups (Figure 4). The winter group exhibited higher complexity (100 links) compared to the summer group (81 links). Moreover, the ratio of positive links to positive links in the summer group was greater than that in the winter group. Several other important network topological properties such as the average degree, network diameter, average path length, and density, also exhibited variations in the network structures (Table 1).

**TABLE 1:** Network indices of the co‐occurrence network of top 30 genera.

Network indices	Summer	Winter
Total nodes	30	27
Total links	81	100
Positive links	69	89
Negative links	12	11
Average degree	5.400	7.407
Network diameter	12.741	13.770
Average path length	2.998	2.838
Density	0.186	0.285

### 3.4 Forage nutrition and its relationships with the gut microbiota

**FIGURE 5:**
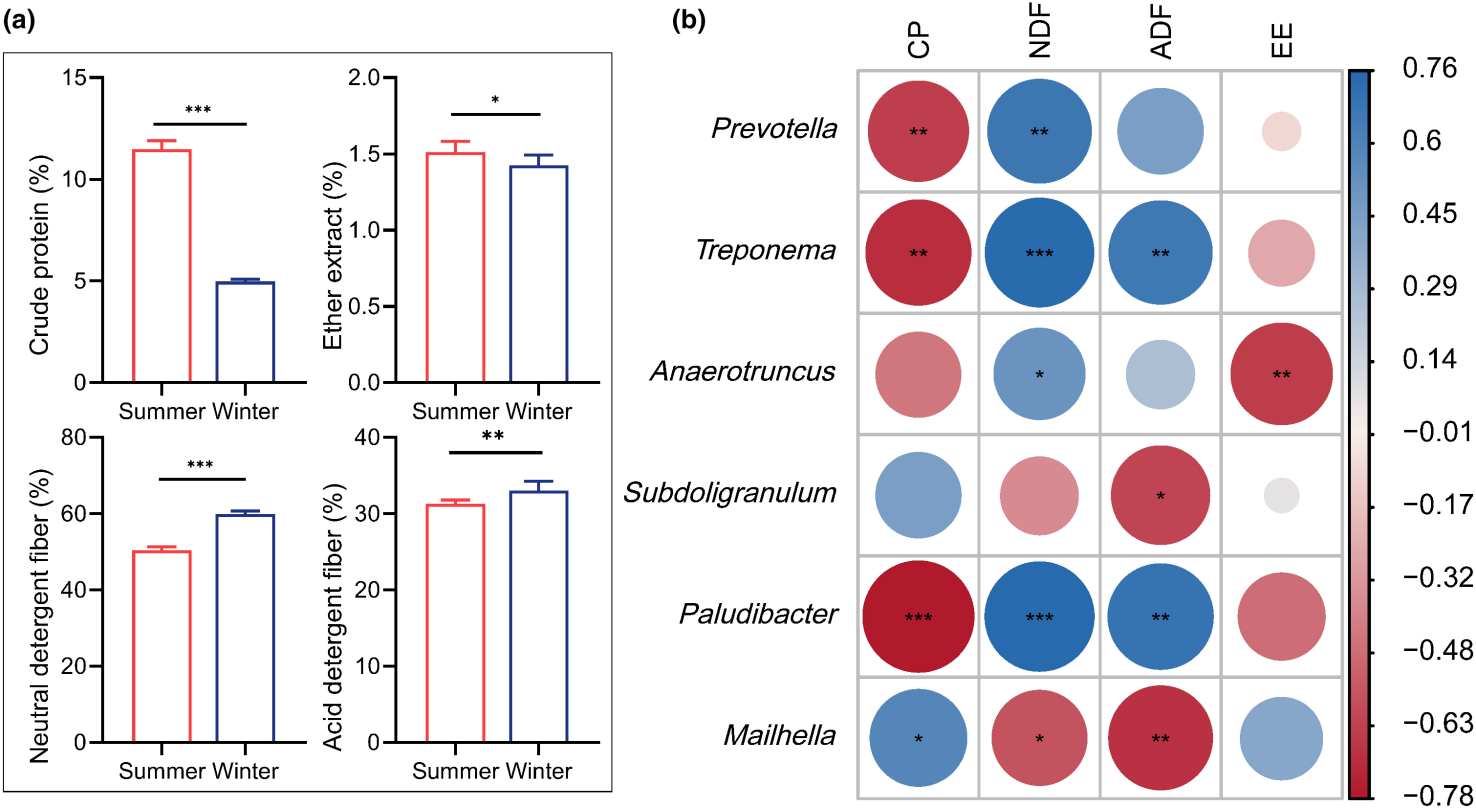
The relationships between forage nutrition and gut microbiota. (a) Forage nutrition between different seasons. (b) Spearman correlations between forage nutrition and dominant genera. Differences are denoted as follows: *p < .05; **p < .01; ***p < .001.

As shown in Figure 5a, the CP and EE content in the summer (CP: 11.45%, EE: 1.53%) were significantly (*p* < .05) higher compared to winter (CP: 4.93%, EE: 1.45%). In contrast, the NDF and ADF content in the winter (NDF: 60.11%, ADF: 33.11%) were significantly (*p* < .05) higher than in the summer (NDF: 50.43%, ADF: 31.57%).

Spearman's correlation analysis was conducted to determine the relationship between the forage nutrition and the relative abundances of the gut microbiota (Figure 5b). The 30 most dominant genera were selected for Spearman's correlation analysis. The content of CP displayed a positive correlation with the relative abundance of the genus *Mailhella*, whereas it exhibited a negative correlation with the relative abundance of the genera *Prevotella*, *Treponema*, and *Paludibacter*. The content of EE was found to have a negative correlation with the relative abundance of the genus *Anaerotruncus*. The abundance of *Prevotella*, *Treponema*, *Anaerotruncus*, and *Paludibacter* genera showed a positive correlation with the NDF content while *Mailhella* displayed a negative correlation. Meanwhile, *Treponema* and *Paludibacter* were positively correlated with the ADF content but *Subdoligranulum* and *Mailhella* showed a negative correlation.

### 3.5 Seasonal differences in gut microbial functions

**FIGURE 6:**
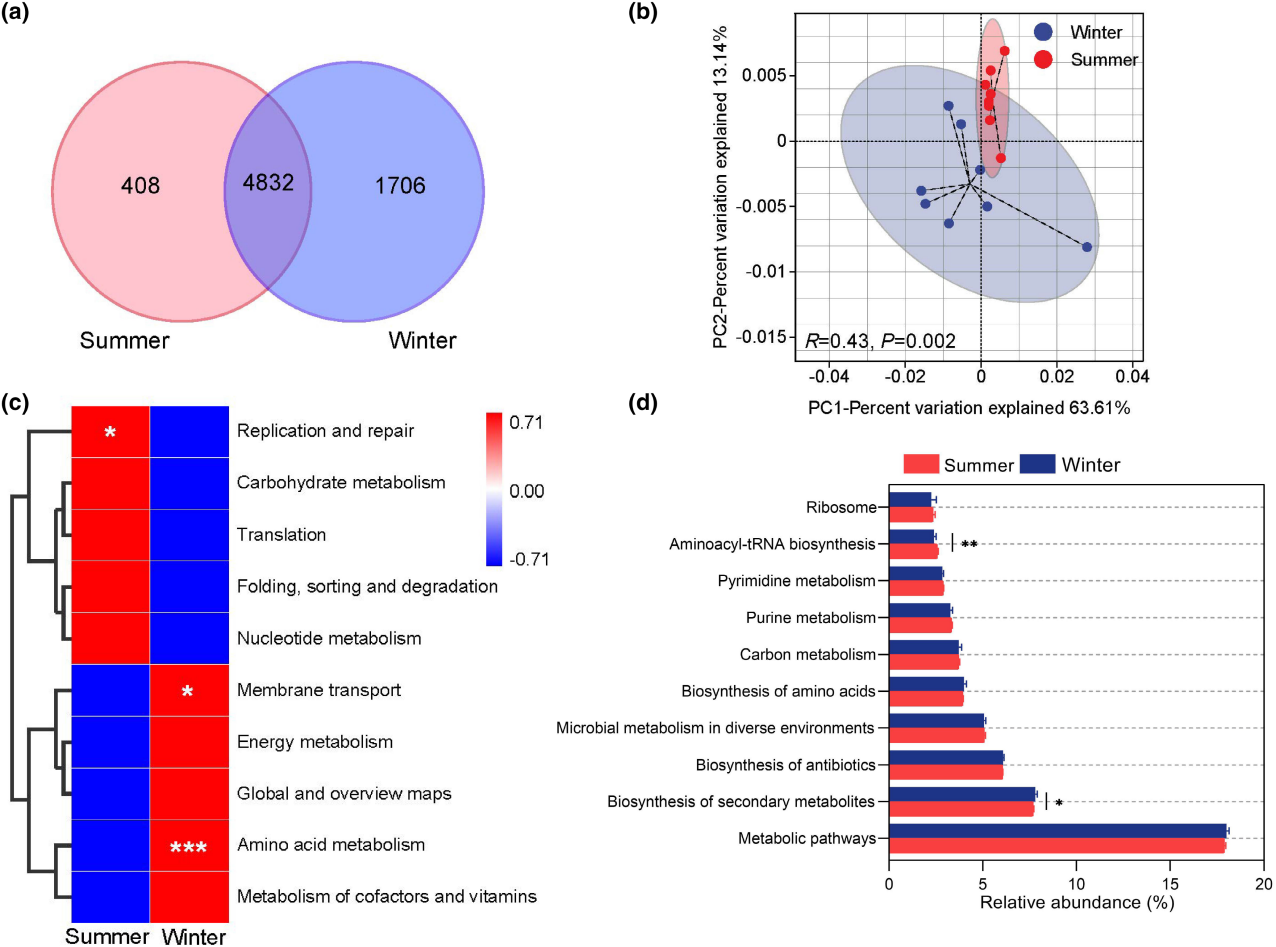
Seasonal variation in gut microbial functions. (a) Venn diagram for distribution of KOs between different seasons. (b) PCoA based on Bray‐Curtis distances of the relative abundance of KOs. (c) Heatmap of gut microbial functions based on KEGG level‐2 pathway. (d) Seasonal differences in relative abundance of KEGG level‐3 pathways. Differences are denoted as follows: *p < .05; **p < .01; ***p < .001.

The gut microbiota of grazing yaks shared a total 4832 KOs between two different seasons, with 408 and 1706 KOs being unique to summer and winter, respectively (Figure 6a). Results from the PCoA showed clear separation of the summer and winter samples based on the Bray‐Curtis distance of the relative abundance of KOs, and the pairwise Adonis test indicated the two groups were significantly different (*R* = 0.43, *p* = .002) (Figure 6b). The clustering heatmap based on the relative abundance of KEGG level‐2 pathways demonstrated a significant enrichment in the replication and repair biological processes in summer (*p* < .05). In contrast, a higher enrichment in genes related to amino acid metabolism (*p* < .001) and membrane transport (*p* < .01) was observed in the winter (Figure 6c). Furthermore, the differences between the groups of the KEGG level‐3 pathways were analyzed, discovering the enrichment of genes on biosynthesis of secondary metabolism in winter, and the aminoacyl‐tRNA biosynthesis in summer (Figure 6d).

**FIGURE 7:**
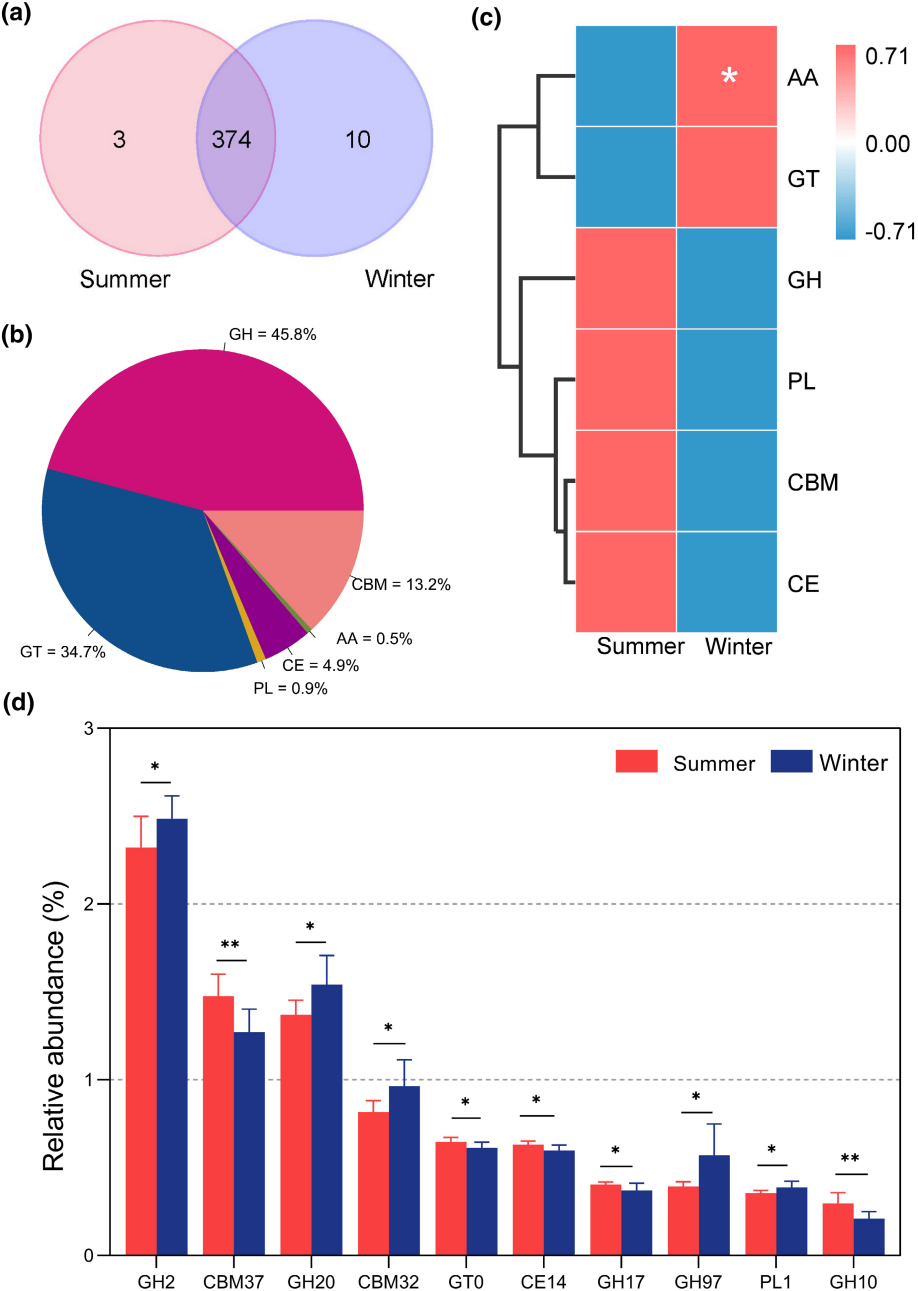
Seasonal variation in gut microbial carbohydrate‐active enzymes (CAZymes). (a) Venn diagram for distribution of CAZymes between different seasons. (b) Distribution of gut microbial CAZymes based on level‐1 (Class). (c) Heatmap of gut microbial CAZymes based on level‐1 (Class). (d) Seasonal differences in relative abundance of level‐2 (Family) CAZymes. Differences are denoted as follows: *p < .05; **p < .01; ***p < .001.

GHs (glycoside hydrolases, 48.8%), GTs (glycoside transferases, 34.7%), CBMs (carbohydrate binding modules, 13.2%), CEs (carbohydrate esterases, 4.9%), PLs (polysaccharide lyases, 0.9%), and AAs (axillary activity enzymes, 0.5%) were annotated (Figure 7b). The gut microbiota of grazing yaks shared 374 CAZymes between summer and winter, and the number of CAZymes unique to summer and winter were 3 and 10 (Figure 7a). The heatmap based on relative abundance of level‐1 (Class) CAZymes showed that the abundance of AAs in winter was significantly higher than that in summer (*p* < .05) (Figure 7c). Furthermore, we analyzed the differences of relative abundance of level‐2 (Family) CAZymes between two groups, and the top 10 significant family CAZymes were shown in Figure 7d. We found that the relative abundance of GH2, GH20, CBM32, GH97, and PL1 were significantly higher (*p* < .05) in the summer group, while CBM37, GT10, CE14, GH17, and GH10 were higher (*p* < .05) in the winter group.

## 4 DISCUSSION

### 4.1 Seasonality of gut microbial diversity

Environmental factor shifts can drive a change in the diversity of gut microbes of animals. For example, changes in the gut microbial diversity and richness were consistent with the seasonal changes of the white‐lipped deer (You et al., 2022) and horseshoe bat (Garay‐Novillo et al., 2019). However, here no significant differences in gut microbial alpha diversity were found between the groups of summer and winter in this study, which may be due to the following reason. All experimental yaks were grazed on the fixed area pasture without feed supplementation from July to December. Although the nutritional quality of the forage varies greatly, there is no difference in the composition of the vegetation community. This finding is consistent with our previous studies that shows that changing nutrient content of the diet under the same food ingredients was not sufficient to cause a strong fluctuation of microbial alpha diversity in yak (Zhang et al., 2021) and Tibetan sheep (Wang et al., 2022). Although there was no notable variance in alpha diversity, a substantial distinction was evident in the gut microbial community structure (beta diversity) between seasons. This finding indicated that seasonal changes have an impact on differences in the microbial community composition of grazing yaks. This phenomenon is common in the seasonal variation of gut microbiota in several animals, such as the white‐headed black langur (Chen et al., 2023), plateau pikas (Fan et al., 2022), and brown frog (Tong et al., 2019). Future research should collect the microbiome of environmental sites to make the association between host gut microbiota and environmental conditions.

### 4.2 Seasonal differences in gut microbial composition

The gut microbiota of yaks is primarily composed of the phyla Firmicutes, Bacteroidetes, Verrucomicrobia, and Proteobacteria, which in accordance with previous studies of ruminants (Liao et al., 2021; Wang, Hu, et al., 2021; Wang, Jin, et al., 2021; Wang, Wu, et al., 2023; Wang, Zhang, et al., 2023). Grazing yaks exhibited a higher abundance of Actinobacteria in winter. Actinobacteria can produce cellulolytic enzymes to degrade complex cellulose (Berlemont & Martiny, 2013). In the winter season, grazing yaks consumed a larger amount of NDF and ADF in their feed. A higher abundance of Actinobacteria can help grazing yaks to degrade crude fiber in forage sources. Furthermore, Actinobacteria was recognized as proficient bioactive natural product producers (Bernal et al., 2015), capable of producing numerous antibiotics and probiotics that play important roles in the disease resistance (L. Fan et al., 2019; Matsui et al., 2012). Therefore, the increased prevalence of Actinobacteria in the gut microbiota in winter is responsible for maintaining gut homeostasis in the face of environmental and dietary changes. Firmicutes assist in breaking down dietary fiber and transforming cellulose to VFAs, thereby enhancing food digestion and nutrient absorption (McAllister et al., 1994). Bacteroidetes primarily facilitate the digestion and absorption of proteins and carbohydrates present in the food (Jami et al., 2014). In this study, an elevated F/B value in winter aids yaks in adapting to their environment, which typically includes food with high crude fiber and low nutrition, allowing them to cope with the cold weather, and this evidence comes from mice (Turnbaugh et al., 2006) and Tibetan wild ass (Gao et al., 2023). At the genus level, *Bacteroides*, *Clostridium*, and *Prevotella* were prominently present in samples from the summer season. *Bacteroides* and *Prevotella*, which belong to the phylum Bacteroidetes, are acknowledge as the primary contributors to carbohydrate metabolism (Karlsson et al., 2011). The white‐headed black langurs, for instance, show an increased abundance of Bacteroidetes (specifically *Bacteroides* and *Prevotella*) as they transition from the dry season to rainy season to facilitate the digestion of simple carbohydrates and proteins found in young leaves (Chen et al., 2023). In this study, the higher proportion of *Bacteroides* and *Prevotella* indicates an adaption to the consumption of large amounts of fresh forage during the summer season.

In the co‐occurrence network analysis of the core microbiota, the summer grazing may stimulate the growth of more native core microbiota. However, severe environmental conditions and low‐nutrient food promoted interactions among the core microbiota in the winter. Previous research has demonstrated that positive links among microbial communities may be unstable and highly responsive to changes in environmental factors (de Vries et al., 2018; Nishida & Ochman, 2021). In addition, the higher ratio of negative to positive correlations in the summer is due to the plants grow luxuriantly in summer and the more abundance food resources. In contrast, the stability of the gut microbiota in grazing yaks is reduced when exposed to complex environmental conditions and changes in diet.

### 4.3 Forage nutrition and its relationships with the gut microbiota

The nutrient composition of pasture is a crucial determinant of the growth performance and health status of grazing livestock (Chapman et al., 2007; Liu et al., 2020). The nutrient composition gradually increases from the greening stage to the grass stage, but with the shift of the season, the pasture gradually yellows and the nutrient content decreases, particularly in winter (Zhao et al., 2018). In the present study, the nutrient content of the forage was significantly correlated with some typical genera of gut microorganisms in grazing yaks. *Prevotella* is a significant genus in the animal gut, playing a crucial role in the breakdown of dietary proteins and carbohydrates (Betancur‐Murillo et al., 2022; Purushe et al., 2010). Studies indicated that a diet abundant in dietary fiber can enhance the abundance of *Treponema*, *Butyrivibrio*, and *Prevotella* in the gut, improve the utilization of cellulose and xylose and encourage the synthesis of short‐chain fatty acids (Le Sciellour et al., 2018; Xie et al., 2018). Notably, there was a negative correlation between EE content and *Anaerotruncus* in this study. It was found that the abundance of *Anaerotruncus* decreased in the gut of mice on a high‐fat diet, which is similar to our finding in yaks. This suggests that *Anaerotruncus* may serve as an indicator genus for intestinal flora disorders and exacerbate obesity (Lai et al., 2018).

### 4.4 Changes in gut microbial function

According to the KEGG database, the relative abundance of genes involved in amino acid metabolism and membrane transport was higher in winter. Increasing evidence suggests that the bacterial amino acid utilization may primarily be for the synthesis of bacterial protein in the small intestine, whereas catabolism dominates amino acid metabolism in the large intestine, particularly under low carbohydrate conditions (Dai et al., 2010; Libao‐Mercado et al., 2009). In response to nutrient restriction and stress, the amino acid metabolism of gut microbiota also play a crucial role in the endurance of gut bacteria under conditions of nutrient scarcity and starvation (Dai et al., 2011). In our study, the gut microbiota in the winter showed an increased amino acid metabolic activity with the aim of enhancing the efficiency of nutrient intake from low protein forages and adapting better to the harsh environment. Besides the amino acid metabolism function, there was a marked increase in the abundance of genes encoding AAs in winter. The AA class comprises enzymes families that participate in breaking down lignin and polysaccharide degradation, such as ligninolytic enzymes and lytic polysaccharide mono‐oxygenases (Levasseur et al., 2013). The lignocellulose content of the pasture is greater in the winter, and the gut microorganisms of the yak exhibit elevated levels of lignin‐degrading enzyme activity to effectuate lignin degradation. Furthermore, the GH2 and GH20 were the two most significant enzymes during the winter season. The families GH2 and GH20, which contain β‐galactosidases, have been shown to have a correlation with mucin‐degrading enzyme activities in the gut microorganisms of yaks (Gong et al., 2020). Host‐secreted mucin glycoproteins are a crucial substrate for gut microorganisms, particularly with the distal gut microbiota (Lin et al., 2023; Yamada et al., 2019). This indicated that grazing yaks can utilize symbiotic relationships between gut microorganisms and mucins secreted by the intestinal wall of the host, thereby achieving more efficient energy expenditure during winter.

## 5 CONCLUSIONS

**FIGURE 8:**
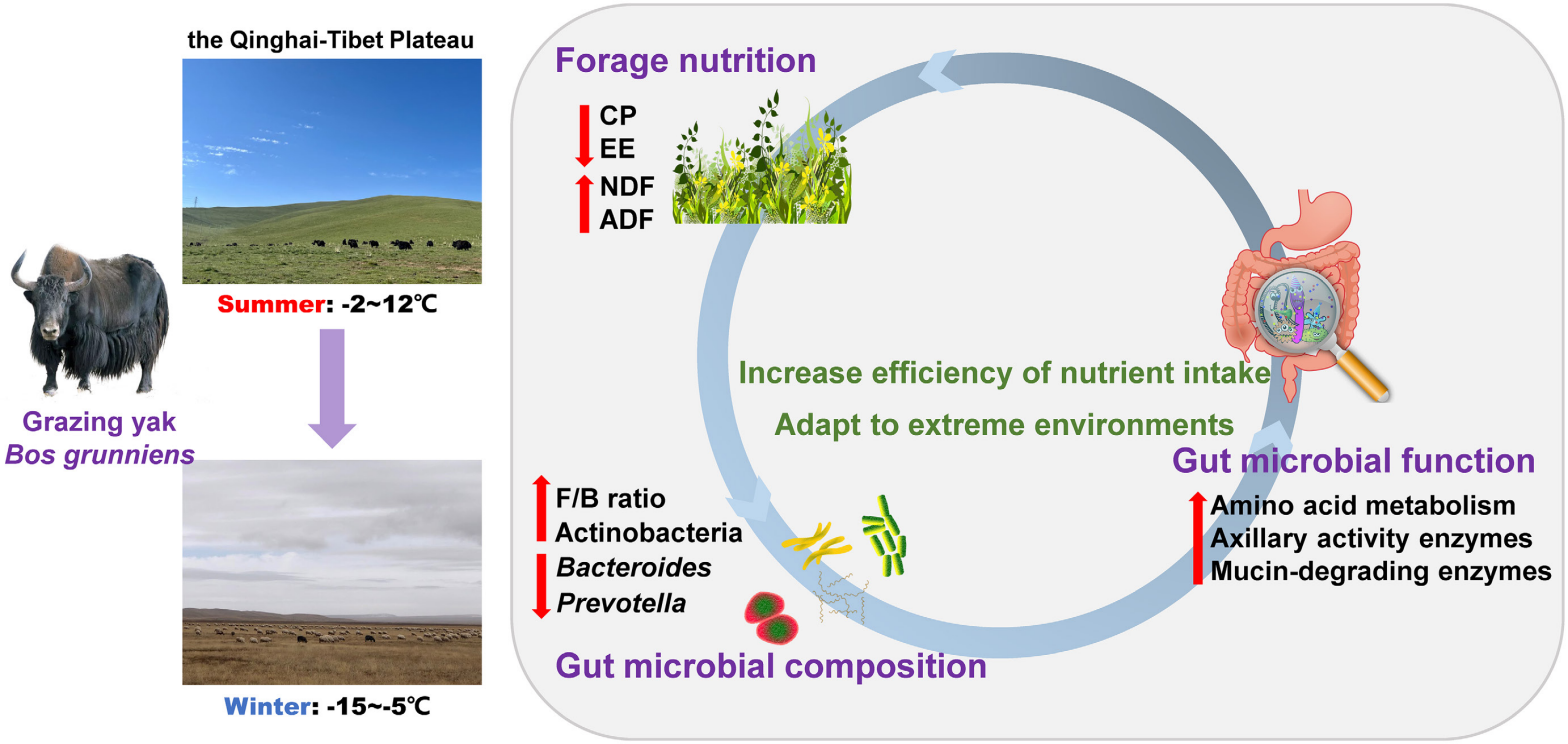
Dynamics of yak gut microbiota in response to seasonal dietary shift.

We found that the gut microbial compositions and functional profile of grazing yaks between two seasons were significantly different. The relative abundance of Actinobacteria and the F/B value were observed to be higher in winter, which may be indicative of the yak' ability to utilize low‐nutrient forages. Forage nutrition exhibits significant seasonal variation, exerting a significant influence on the composition of the gut microbiota. Metagenomic analysis revealed that the functional pathways and CAZymes were also different between the seasons. These variations in composition and function were closely related to the adaption of yaks to the harsh environment in winter (Figure 8). These efforts provide novel insights into the mechanisms of highland animal adaptation to external environment from the perspective of the gut microbiome. In subsequent studies, experiments can be carried out to isolate and transplant bacteria to verify the biological functions of the gut microbiota.

## AUTHOR CONTRIBUTIONS

**Xungang Wang:** Conceptualization (lead); data curation (lead); investigation (equal); visualization (equal); writing – original draft (lead); writing – review and editing (lead). **Tongqing Guo:** Data curation (equal); methodology (equal); resources (equal). **Qian Zhang:** Methodology (equal); resources (equal); software (equal); validation (equal). **Na Zhao:** Data curation (equal); software (equal); validation (equal). **Linyong Hu:** Methodology (equal); software (equal); validation (equal); visualization (equal). **Hongjin Liu:** Data curation (equal); investigation (equal); validation (equal); visualization (equal). **Shixiao Xu:** Conceptualization (equal); funding acquisition (supporting); project administration (equal); writing – review and editing (lead).

## FUNDING INFORMATION

This research was funded by the National Key Research and Development Program of China (2021YFD1600200), the Chief Scientist Program of Qinghai Province (2024‐SF‐102), the Joint fund project of NSFC (U21A20250), and the Joint Special Project of Sanjiangyuan National Park (LHZX‐2022‐02).

## CONFLICT OF INTEREST STATEMENT

The authors declare no competing interests.

## Supporting information


Table S1.

Table S2.

## Data Availability

Raw sequence data have been submitted to the Genome Sequence Archive (GSA) in National Genomics Data Center (https://ngdc.cncb.ac.cn/gsa) under accession number CRA013457.
