# Supplementary material for: Seasonal variations in composition and function of gut microbiota in grazing yaks: Implications for adaptation to dietary shift on the Qinghai‐Tibet plateau
Source: Ecol Evol. 2024 Oct 22;14(10):e70337. doi: 10.1002/ece3.70337 (PMC11495855; doi:10.1002/ece3.70337)
Supplement: Supplementary file 1 — Table S1. [file ECE3-14-e70337-s001.docx]

TABLE S1. Metagenome sequence statistics in this study

| Items | Summer | Winter | SEM | *P*-value |
| --- | --- | --- | --- | --- |
| Clean data base/ bp | 10 567 756 861 | 10 769 849 383 | 135 024 448 | 0.530 |
| No host clean data base/ bp | 10 270 398 304 | 8 954 478 878 | 352 671 439 | 0.077 |
| Reads number | 68 883 988 | 60 115 293 | 2 332 833 | 0.077 |
| Contig number | 1 181 859 | 902 964 | 73 318 | 0.094 |
| Gene number after prediction | 1 431 586 | 1 078 718 | 102 628 | 0.134 |
